# De novo design of pH-responsive self-assembling helical protein filaments

**DOI:** 10.1038/s41565-024-01641-1

**Published:** 2024-04-03

**Authors:** Hao Shen, Eric M. Lynch, Susrut Akkineni, Joseph L. Watson, Justin Decarreau, Neville P. Bethel, Issa Benna, William Sheffler, Daniel Farrell, Frank DiMaio, Emmanuel Derivery, James J. De Yoreo, Justin Kollman, David Baker

**Affiliations:** 1 Department of Biochemistry, University of Washington, Seattle, WA USA; 2 Institute for Protein Design, University of Washington, Seattle, WA USA; 3 Department of Materials Science and Engineering, University of Washington, Seattle, WA USA; 4 Physical Sciences Division, Pacific Northwest National Laboratory, Richland, WA USA; 5 MRC Laboratory of Molecular Biology, Cambridge, UK; 6 Department of Bioengineering, University of Washington, Seattle, WA USA; 7 Howard Hughes Medical Institute, University of Washington, Seattle, WA USA

**Keywords:** Biomaterials, Biomaterials, Nanostructures

## Abstract

Biological evolution has led to precise and dynamic nanostructures that reconfigure in response to pH and other environmental conditions. However, designing micrometre-scale protein nanostructures that are environmentally responsive remains a challenge. Here we describe the de novo design of pH-responsive protein filaments built from subunits containing six or nine buried histidine residues that assemble into micrometre-scale, well-ordered fibres at neutral pH. The cryogenic electron microscopy structure of an optimized design is nearly identical to the computational design model for both the subunit internal geometry and the subunit packing into the fibre. Electron, fluorescent and atomic force microscopy characterization reveal a sharp and reversible transition from assembled to disassembled fibres over 0.3 pH units, and rapid fibre disassembly in less than 1 s following a drop in pH. The midpoint of the transition can be tuned by modulating buried histidine-containing hydrogen bond networks. Computational protein design thus provides a route to creating unbound nanomaterials that rapidly respond to small pH changes.

## Abstract (web summary)

Engineering the tunability of protein assembly in response to pH changes within a narrow range is challenging. Here the authors report the de novo computational design of pH-responsive protein filaments that exhibit rapid, precise, tunable and reversible assembly and disassembly triggered by small pH changes.

## Main

Nature uses pH to control protein assembly. For example, spider silk protein has a pH-sensitive relay to control the generation of solid silk^1^, CTP synthase filaments polymerize at low pH to facilitate homeostasis during budding yeast starvation^2^ and R bodies generate force in response to pH changes by undergoing local conformational changes that propagate through an extended lattice of identical subunits^3^. These materials have inspired bioengineers to try to design pH-dependent protein materials. Designed pH-responsive proteins are a promising new class of such materials with potential applications in fields such as tissue engineering, drug and gene delivery and self-healing biomaterials. Progress has been made in producing pH-responsive assembling nanomaterials by mimicking the silk protein domain^4^ and introducing histidine residues by staggering extended interaction motifs between peptide coiled coils^5^. However, the de novo design of pH-responsive filaments with precisely defined structures and tunable pH transition points is an unmet challenge.

We reasoned that this challenge could be overcome by the computational design of self-assembling protein filaments from subunits containing multiple buried residues that switch their protonation state over a small range of pH and drive unfolding/folding transitions. As a designed filament would contain hundreds or more of such subunits, the environmental response to even small pH changes should be extremely sensitive and cooperative. Attractive candidates include previously designed pH-dependent trimeric helical bundles that undergo a very sharp unfolding transition at a tunable pH between 4.0 and 6.5 with nine buried histidines in buried hydrogen networks^6^, but the internal symmetry is not compatible with incorporation into an extended filament with general helical symmetry as it would lead to large-scale bundling of different fibres.

## Results

### Designing pH-responsive fibres

**Fig. 1:**
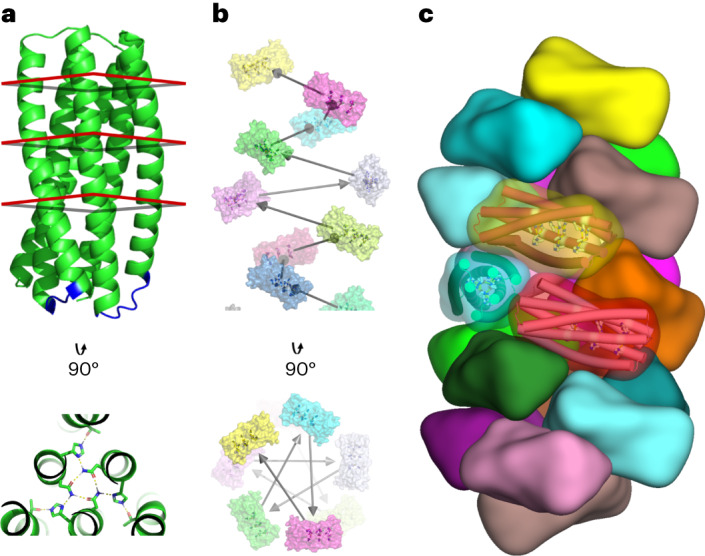
Design strategy for generating pH-dependent protein filaments. **a**, Top: pH-dependent protein trimers are converted into monomers by addition of short loops connecting the subunits (blue). The connected monomers contain two or three extended hydrogen bond networks, each involving three buried histidine residues. The positions of the hydrogen bond networks are indicated by the red squares. Bottom: the top-down view shows the extensive hydrogen bond network with three central asparagines coordinated by histidine residues, which accept hydrogen bonds from the asparagines and donate hydrogen bonds to threonines on the outer helices. As each histidine makes two hydrogen bonds, protonation completely disrupts the networks. **b**, Computational sampling of alternative helical packing arrangements of subunits shown in **a** viewed from the side (top) and from above (bottom). Each subunit is shown in a different colour. The black arrows represent the rigid body transform that generates the helical assembly. **c**, Compact packing arrangement of fibre subunits in the lowest energy assemblies following sequence design. The helices are shown as rods and the pH-sensitive hydrogen bond networks are shown as sticks. Each subunit is shown in a different colour.

**Fig. 2:**
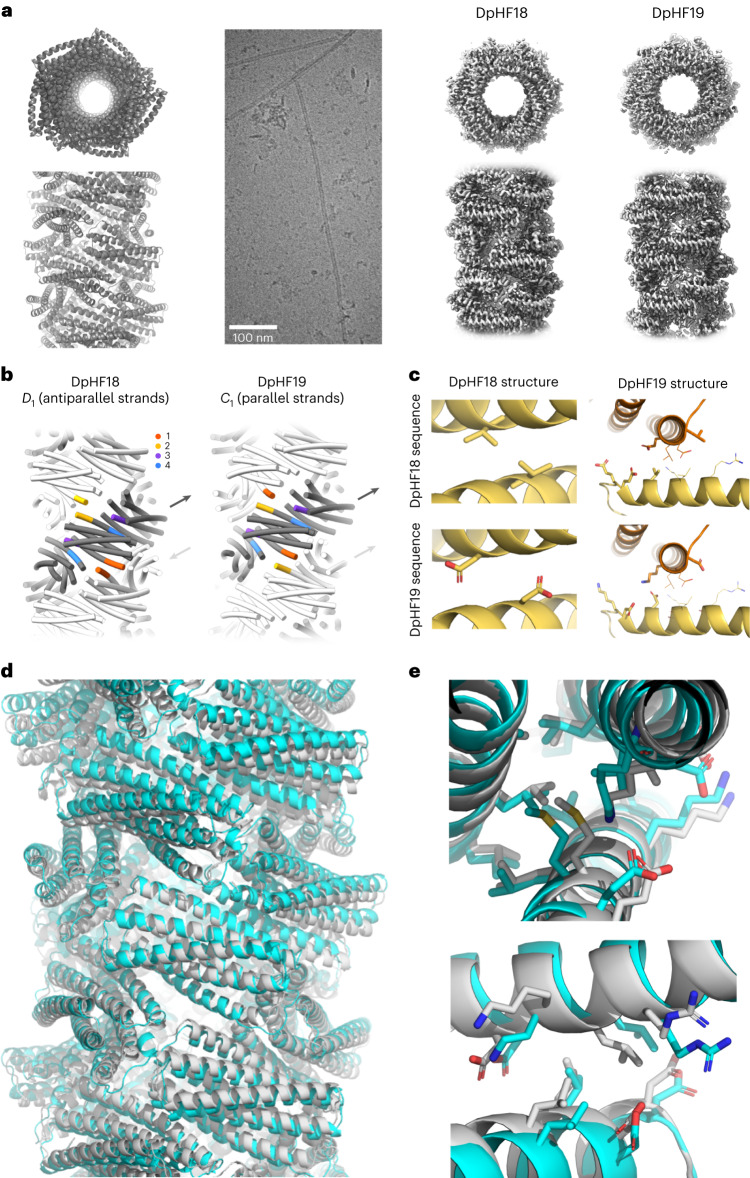
Cryo-EM structural characterization of designed filaments. **a**, Computational design model (first column) and cryo-EM maps of DpHF18 (third column, at 3.6 Å resolution) and DpHF19 (fourth column, at 3.4 Å resolution) viewed from above (top) and from the side (bottom). The second column shows representative filaments in a cryo-EM micrograph. **b**, Comparison of the antiparallel structure of DpHF18 with *D*_1_ symmetry (left) and the redesigned DpHF19 structure with the original helical symmetry (right). Interfaces 1–4 are shown in different colours (see the legend). Arrows indicate the directionality of the antiparallel or parallel strands. **c**, Positions of the architecture switching substitutions. The original (top) and redesigned interface (bottom) sequences in DpHF18 and DpHF19 are modelled on both backbone structures. The five substitutions introduced during refinement are shown as thicker sticks, and the relevant surrounding residues as thinner lines. The unintended helix packing in the DpHF18 structure is disfavoured in the redesign by substituting a valine residue and a leucine residue (shown in Supplementary Fig. 3) for aspartates (left column). Three additional substitutions introduce hydrogen bond networks across the desired interface (right column). **d**,**e**, The cryo-EM structure of DpHF19 (cyan) is nearly identical to the computational design model (grey) (**d**) in helical architecture (**e**), with a C-alpha r.m.s.d. of 1.4 Å between interface residues. The main and second interface are shown at the top and the bottom.

The pH-dependent trimers disassemble over a very narrow pH range with high cooperativity that results from the many histidine residues simultaneously being protonated/deprotonated. The pH of the transition midpoint and the cooperativity can be tuned between 4.0 and 6.5 by modulating the strength of the inter-subunit interactions and varying the number of histidines. To break the internal symmetry of the trimer to prevent bundling and allow the design of asymmetric interfaces to drive fibre assembly, we designed short loops to connect the three protomers in one of the trimers, called pRO-2.3, which contains six buried histidines (PDB ID: 6MSQ) into a single chain (Fig. 1a). We then applied the filament design method described in Shen et al.^7^ to dock these connected monomers into a wide variety of helical filament arrangements and design the amino acid residues at the newly formed interfaces to drive assembly (Fig. 1b,c). We generated 45,000 helical filament backbones and selected 18 designs for experimental testing on the basis of the predicted energy of fibre formation and other metrics (Methods). The designs (which we refer to as de novo-designed pH-responsive helical filaments (DpHFs)) were expressed in *Escherichia coli* and purified (Methods). All 18 designs were well expressed and soluble. Two designs (DpHF7 and DpHF18) were found to form filaments, as seen in negative stain electron microscopy (EM) images (Supplementary Fig. 1A and Fig. 2 top left).

### Structural characterization

We used cryogenic EM (cryo-EM) to determine the structures of DpHF7 and DpHF18 at high resolution. The DpHF18 subunit structure and the primary (largest) interface between subunits in the filament are very similar in the cryo-EM structure and computational model. However, the second small hetero interface deviates from the design model, resulting in antiparallel dihedral symmetry (*D*_1_) in addition to the helical symmetry generated by the main interface (Fig. 2a,b). We carried out a second round of sequence design to explicitly favour the designed fibre packing arrangement over this alternative, and identified five substitutions to the charged residues at the second interface. Two substitutions (V29D and L82D) knocked out the *D*_1_ interface (Fig. 2c (left) and Supplementary Fig. 3) and the other three substitutions (E32D, E33K and E87K) favoured the originally designed interface (Fig. 2c (right)). This redesign, denoted DpHF19, readily formed filaments, and the cryo-EM structure at 3.4 Å revealed that the structure was very close to the design model (1.4 Å root mean squared deviation (r.m.s.d.) over interface residues; Fig. 2a,d,e) with pure helical symmetry and no additional dihedral symmetry (Fig. 2b). The N terminus of the subunit faces the inside of the pore with the N-terminal helix interacting with two adjacent subunits, the C-terminal helix interacts with one of these subunits and the C terminus faces a loop from a third subunit. The helical symmetry of DpHF19 is one-start, relating non-contacting subunits with a rise of 8.4 Å and rotation of −148.9°; in cross-section five subunits form a ring (Supplementary Fig. 4), with inter-subunit rigid body transforms as indicated in Fig. 1b. The resulting overall architecture can be viewed as a right-handed helix composed of two identical, parallel strands with contacting subunits (Fig. 2b). In contrast with the near-perfect agreement of the DpHF19 design model and the cryo-EM structure, the structure of DpHF7 differs considerably from the corresponding design model (Supplementary Fig. 5).

### Characterization of pH-response

To determine whether the pH transition midpoint could be modulated by design, we made a version with three additional buried histidine residues (bringing the total to nine), referred to as DpHF19_9his hereafter. The originally designed non-filament-forming trimer pRO-2 with the same buried nine histidines had a disassembly pH of 5.5 (ref. ^6^). DpHF19_9his assembled into fibres very similar to the six-histidine version (Supplementary Fig. 2, bottom left).

**Fig. 3:**
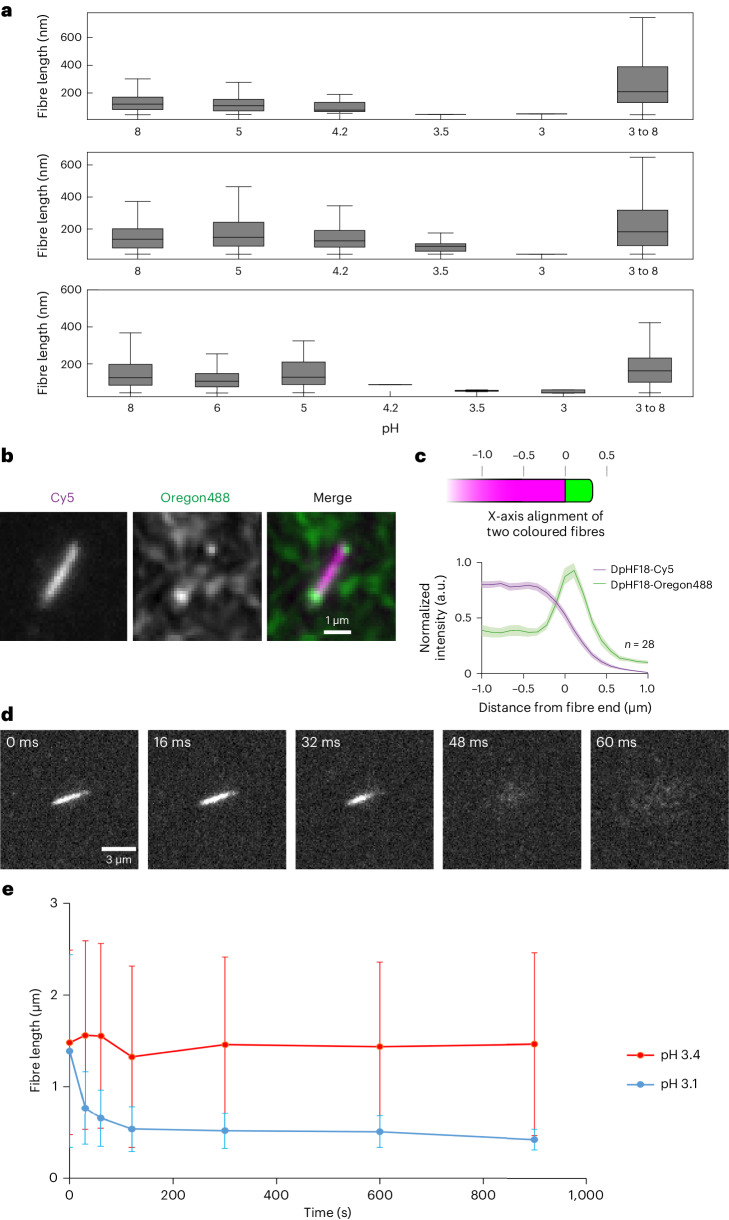
Characterization of fibre assembly and disassembly by EM and fluorescence microscopy. **a**, Fibre length distributions quantified from negative stain EM micrographs of DpHF18 (top), DpHF19 (middle) and DpHF19_9his (bottom) in pH 8, 6, 5, 4.2, 3.5 and 3 buffer, and after incubation at pH 3 followed by raising the pH to 8. In the box and whisker plots, the centre line is the median, the box represents Q1 to Q3, the lower whisker is Q1–1.5 × IQR and the upper whisker is Q3 + 1.5 × IQR (Q1 is the first quartile, Q3 is the third quartile and IQR is the interquartile range). From left to right, the numbers of independent fibres quantified for DpHF18 are 436, 329, 3, 1, 1, 166; for DpHF19, 61, 1491, 207, 26, 1, 33; and for DpHF19_9his, 427, 522, 26, 1, 2, 9, 155. **b**,**c**, TIRFM image (**b**) and Cy5 and Oregon488 fluorescence intensity line scans (**c**) (mean ± s.e.m.) of pre-assembled DpHF18–Cy5 fibres elongated with DpHF18–Oregon488 monomers quantified from 21 independent fibres. A schematic depicting how the line scans in the plot were aligned is shown above. **d**, Rapid disassembly of a DpHF18–Cy5 fibre when the pH dropped from 8 to 3 at the time points indicated. **e**, Length of DpHF18–Cy5 fibres as a function of time following a drop in pH from 8.0 to 3.4 or 3.1, followed by fluorescence microscopy. Data are presented as mean ± s.d. From left to right, the plot represents 510, 511, 414, 695, 563, 500 and 496 independent fibre objects at pH 3.4, and 698, 1,090, 466, 427, 113, 31 and 13 at pH 3.1. Fibres remain assembled at pH 3.4 but disassemble at pH 3.1, highlighting the sharp pH dependence of assembly.

We first characterized the pH response of the designed filaments using negative stain EM. DpHF18, DpHF19 and DpHF19_9his were incubated in buffer at pH 8, which was then reduced to pH 6, 5, 4.2, 3.5 or 3 through the addition of citric acid and then raised back from 3 to 8 by adding 1 M Tris-HCl at pH 8. Fibre lengths were quantified from the negative stain images using the filament tracer method in cryoSPARC^8^ (Fig. 3a and Supplementary Fig. 2). DpHF18, DpHF19 and DpHF19_9his disassembled at pH 3.5, 3 and 4.2, respectively, and all reformed when the pH was raised from 3 back to 8. The pH transition is thus reversible, and the pH transition midpoint can be increased by increasing the number of buried histidines. DpHF7 also has a reversible pH response, with disassembly at pH 4 and reassembly at pH 8 (Supplementary Fig. 1).

To characterize the pH-induced conformational changes in real time, we labelled DpHF18 by cysteine conjugation with sulfo-Cy5 maleimide or Oregon488 dyes. We first used total internal reflection fluorescent microscopy (TIRFM) to monitor filament assembly. Pre-assembled DpHF18–Cy5 fibres were mixed with freshly disassembled DpHF18–Oregon488 monomers at pH 8, and growth of Oregon488-labelled fibres was observed at both tips of the Cy5-labelled seed fibres (Fig. 3b,c), indicating that the fibres grow from their ends.

To study the dynamics of DpHF18 filament disassembly, we assembled DpHF18–Cy5 fibres and reduced the pH from 8 to 3 in a flow cell while imaging with TIRFM. We observed disassembly in less than 1 s after the pH drop (Fig. 3d and Supplementary Fig. 6). To probe the pH response sensitivity, we compared the disassembly of DpHF18–Cy5 at pH 3.1 and 3.4 using cell profiler^9^ to track the change in the lengths of fibres. The fibre lengths dropped very fast at pH 3.1, while remaining stable at pH 3.4, demonstrating a sharp pH transition over 0.3 pH units (Fig. 3e and Supplementary Fig. 7).

**Fig. 4:**
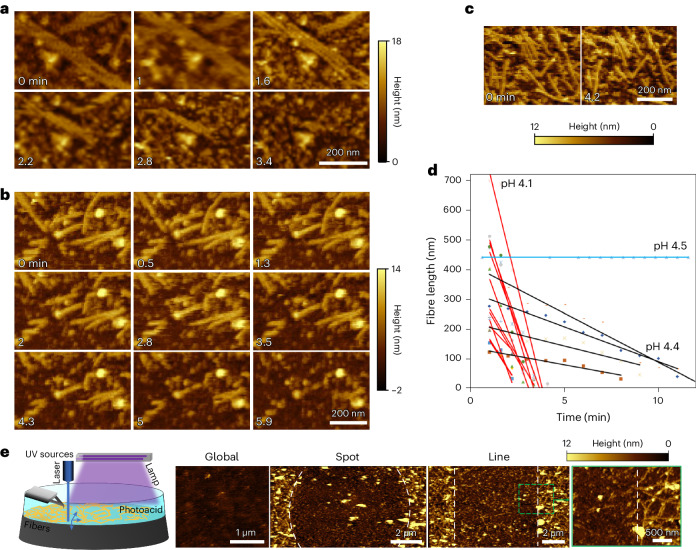
Dynamics of fibre disassembly monitored via liquid-phase AFM. **a**–**c**, Time-lapse of AFM images of DpHF19_9his fibres when the pH is reduced from 8 to 4.1 (**a**), 4.4 (**b**) and 4.5 (**c**). 0 min is defined as the time when the acidic solution is introduced into the flow cell. **d**, Comparison of linear-fit fibre disassembly rates at pH 4.1, 4.4 and 4.5. Fibre length is a measurement of the longest remaining fibre fragment. **e**, External stimulus-driven fibre disassembly. As illustrated in the diagram on the left, local (405 nm laser) and global (364 nm lamp) activation of photoacids lowers the solution pH for the disassembly of fibres on a surface. The location, pattern and area can be precisely controlled by the spot size of the UV light source. The image with a green outline shows a higher magnification of the area in the green dashed rectangle. White dashed lines delineate areas where fibres were exposed and disassembled.

To monitor the pH response kinetics and disassembly mechanism of DpHF19_9his at higher spatial resolution, we used liquid-phase atomic force microscopy (AFM). We monitored the lengths of individual fibres (weakly immobilized on poly-lysine coated mica) when the buffer pH was changed from 8 to 4.1, 4.4, 4.5 (Fig. 4a–c) or 4.7^7,10^. On average, the initial fibre length decreased fastest at pH 4.1 (108 ± 64 nm/min) followed by 4.4 (21 ± 9 nm/min), while no significant change was observed at pH 4.5 or above (Fig. 4d). There is thus a sharp transition for disassembly over 0.1 pH units (from 4.5 to 4.4) and a steep increase in disassembly kinetics over 0.3 pH units (from 4.4 to 4.1).

To understand the disassembly mechanism of an individual fibre, we further analysed the change in length from the two ends of individual fibres to the fibre centres (half of the full fibre length) (Supplementary Fig. 8). This revealed that there are multiple mechanisms for disassembly; the events are clearly observable at pH 4.4 due to slower kinetics. For most fibres, the disassembly rates were the same on both ends, but for some, disassembly at one of the ends was slower, probably due to the transient binding of the fibre end to the surface (poly-lysine coated mica). Fibres can also fragment, leading to a large decrease in the initial fibre length, especially for longer fibres; the fragments then disassemble from both ends or just one end, as seen for the full fibres.

To harness the pH response mechanism and introduce an additional layer of external stimulus-driven response, we used a pH 5.5 buffer containing a photoacid (2-nitrobenzaldehyde) as the solvent/imaging medium for AFM^11^. Activation of the photoacid with a UV source, with global (364 nm lamp) or local (405 nm laser) exposure, released protons locally, reducing the solution pH. AFM characterization showed that global exposure disassembled fibres across the whole exposed surface, whereas local exposure dissolved small patches of fibres to generate spot or line patterns of disassembled fibres (Fig. 4e). In contrast, control experiments on deposited fibres—using solutions without photoacid, solutions with photoacid but overexposed to the high-power laser and solutions with consumed photoacid after patterning—demonstrated negligible fibre disassembly on the surface (Supplementary Fig. 9), strongly suggesting that photoacid illumination drives disassembly by (as expected) reducing the local solution pH.

### Limitations

There is still considerable room for improvement in our approach. Even small deviations in the geometry of the designed interfaces from the fibre design model will compound as the fibre propagates and can lead to non-specific aggregation or alterations in fibre geometry, as observed for the DpHF18 design. Recently developed deep learning protein design methods^12,13^ could increase the accuracy of interface design and hence increase the success rate. On the experimental side, determining the structure of early intermediates in fibre assembly and disassembly remains extremely challenging. The development of a new generation of graphene-based liquid cells and highly sensitive direct electron detectors may enable imaging of such intermediates in the near future via liquid-phase transmission electron microscopy.

## Conclusion

The ability to generate micrometre-scale pH-responsive filaments is an advance in the computational design of environmentally responsive protein nanomaterials, and demonstrates that de novo design can now create material properties that are difficult to achieve by traditional protein engineering or by mimicking native pH-responsive protein assembly. The two fibres systems described here exhibit remarkable and tunable pH dependence of disassembly: DpHF18–Cy5 remained assembled at pH 3.4 but disassembled at pH 3.1 (Fig. 3e), whereas DpHF19_9his was assembled at pH 4.5 but disassembled at pH 4.4 (Fig. 4b–d). The sharpness of the pH dependence probably arises from the very large number of protonatable groups: each subunit contained six (DpHF18 and DpHF19) or nine (DpHF19_9his) buried histidines, and the fibres contained hundreds of subunits. The close packing of the subunits considerably reduced the pH of fibre disassembly below that of the monomers (pH 4.5 and 5.5, respectively^6^), and made disassembly highly cooperative—we observed little change in fibre length for both systems just above the pH transition point. Hydrogels and other higher-order materials incorporating our designed fibres should inherit their pH dependence, providing new routes for the environmentally triggered release of embedded components (monomeric proteins, peptides or small molecules) for drug delivery and other applications. More generally, the ability to precisely design reconfigurable unbounded protein systems with atomic-scale resolution opens new opportunities to create sophisticated environmentally sensitive nanomaterials.

## Methods

### Computational design strategy

Short loops to connect pRO-2.3 helices into a single chain were designed using an exhaustive database of backbone samples composed of fragments spanning two helical regions as identified by DSSP in high-resolution crystallographic structures (as described previously^14^). Loops were identified in this database via rigid alignment of the terminal residues of the fragment and target using an optimized superposition algorithm^15^. Candidates that met an alignment tolerance of 0.35 Å RMSD were aligned to the target backbone via torsion–space coordinates and soft coordinate constraints to the aligned candidate backbone heavy-atom coordinates. Candidate loop sequences were then designed under sequence profile constraints generated via alignment of the loop backbone to the source structure database. The candidates with the lowest scores were selected for the final loop design.

Helical docking and design methods^7^ were applied to the linked pRO-2.3 to generate helical filament design models. The following criteria filtered individual design trajectories: a discrepancy exceeding −15.0 Rosetta energy units between the bound (polymeric) and unbound (monomeric) states, an interface surface area surpassing 700 Å^2^, a Rosetta shape complementarity exceeding 0.62 and an unsatisfied polar residues count below 5. Designs satisfying these criteria underwent manual refinement, involving single-point reversions to mutations deemed non-contributory to stabilizing the interface’s bound state. The top-scoring design for each docked configuration was then integrated into a finalized protein set for experimental validation.

### Protein expression and purification

The synthetic genes for a total of 18 designs were optimized for expression in *Escherichia coli* and acquired from IDT, then inserted into the pET29b+ vector’s multiple cloning site between NdeI and XhoI restriction sites. These constructs were introduced into BL21* (DE3) *E. coli* competent cells. Transformants were cultured in 50 ml Terrific Broth medium supplemented with 200 mg l^−1^ kanamycin. Expression, under the control of a T7 promoter, proceeded for 24 h at 37 °C using Studier autoinduction^16^ until cultures were harvested by centrifugation. Cell pellets were resuspended in Tris-buffered saline (TBS) and lysed with Bugbuster detergent. The soluble fraction, clarified by centrifugation, underwent purification via Ni^2+^ immobilized metal affinity chromatography using Ni-NTA Superflow resin. The resin with bound cell lysate was washed with ten column volumes of 40 mM imidazole and 500 mM NaCl, followed by elution with 400 mM imidazole and 75 mM NaCl. The soluble and insoluble fractions were subjected to SDS–polyacrylamide gel electrophoresis analysis. Samples exhibiting protein bands at the correct molecular weight were chosen for electron microscopy screening. Selected designs were scaled up to 0.5 l for further characterization, with expression again proceeding for 24 h at 37 °C using Studier autoinduction^16^ before harvesting by centrifugation. Cell pellets were resuspended in TBS and lysed by microfluidization, followed by purification as described above.

### Negative stain EM

Soluble fractions were concentrated in TBS (25 mM Tris buffer, 75 mM NaCl, pH 8) for electron microscopy screening. A 6 µl droplet (1 µl sample instantly diluted with 5 µl of buffer) was applied onto negatively glow-discharged, carbon-coated 200-mesh copper grids, washed with Milli-Q water and stained using either 0.75% uranyl formate (pH 4.0) or Nano-W (pH 6.8) purchased from Nanoprobes, Inc. as described previously^17^. Screening was conducted using either a 100 kV Morgagni M268 transmission electron microscope (FEI) or a 120 kV Talos L120C transmission electron microscope (ThermoFisher). Images were captured using a bottom-mount Teitz CMOS 4k camera system and processed for enhanced contrast using Fiji software (version: 2.14.0/1.54f)^18^ for clarity.

Fibre lengths were quantified using the fibre tracing algorithm in cryoSPARC^8^. This method identifies fibres by cross-correlation to a template class and tracing contiguous fibres from the identified particles. A template class generated from DpHF19 was used for all fibres measured. Fibres were filtered according to the average curvature (<0.0005 Å^−1^) and the average normalized cross-correlation (>0.5) across each fibre. For DpHF18, we used 5, 2, 3, 20, 28 and 21 images for pH 3, 3.5, 4.2, 5, 8 and 3 to 8, respectively. For DpHF19, we used 7, 8, 8, 28, 4 and 5 images for pH 3, 3.5, 4.2, 5, 8 and 3 to 8, respectively. For DpHF19_9his, we used 6, 6, 8, 14, 15, 8 and 4 images were used for pH 3, 3.5, 4.2, 5, 6, 8 and 3 to 8, respectively.

### Cryo-EM

Cryo-EM samples were prepared by applying protein to CFLAT holey-carbon grids, blotting away liquid and plunging the grids into liquid ethane using a Vitrobot (ThermoFisher). For DpHF19, videos were acquired on a Glacios microscope (ThermoFisher) equipped with a K-2 Summit Direct Detect camera (Gatan Inc.) operating in counting mode, with a pixel size of 1.16 Å per pixel, 50 frames and a total electron dose of 65 Å^−^^2^. For DpHF18 and DpHF7, videos were acquired on a Titan Krios (ThermoFisher) equipped with a K-2 Summit Direct Detect camera (Gatan Inc.) operating in super-resolution mode, with a pixel size of 0.525 Å per pixel, 50 frames and a total electron dose of 90 Å^−^^2^. Automated data collection was performed using Leginon^19^ version 3.4. Data processing was performed using cryoSPARC^8^, and workflows are summarized in Supplementary Figs. 10–12. The videos were aligned by patch motion correction, with super-resolution videos binned to a pixel size of 1.05 Å. Contrast transfer function (CTF) parameters were estimated using patch CTF. Template-free filament tracing was performed on a subset of images, and the resulting particles were subjected to 2D classification. Selected 2D classes were then used as templates for template-based filament tracing on full datasets. Following multiple rounds of 2D classification, selected particles were subjected to 3D refinement with helical symmetry imposed and non-uniform refinement enabled. For DpHF19, we imposed one-start helical symmetry relating individual, non-contacting subunits, rather than the two-start helical symmetry parameters. For DpHF7 and DpHF19, per-particle defocus, beam tilt and spherical aberration were also refined. Density modification was performed using ResolveCryoEM in Phenix^20,21^ version phenix-1.20.1. Atomic models for DpHF18 and DpHF19 were refined into cryo-EM maps using ISOLDE^22^, followed by real-space refinement in Phenix, with rotamer and Ramachandran restraints disabled and with reference restraints imposed by the input starting model. The elucidation of the model for DpHF7 employed the de novo model building protocol on the segmented cryo-EM asymmetric unit density^23^. Subsequent residue incorporation and refinement were achieved using RosettaCM^24^ version 2019.31, leveraging symmetry across the unsegmented cryo-EM map for optimal fit-to-density and intra-filament interfaces. A final round of real-space refinement was performed in Phenix, as described above for DpHF18 and DpHF19. Cryo-EM data collection, refinement and validation statistics are summarized in Supplementary Table 1.

### TIRFM

#### Fibre assembly

To image seeded nucleation of pH-responsive fibres, DpHF18 fibres were labelled with two different maleimide-conjugated fluorophores, Oregon488 and sulfo-Cy5. Fibres were labelled with a 10× molar excess, in PBS + 1 mM TCEP for 4 h at room temperature, before buffer exchange into TBS (25 mM Tris, 100 mM NaCl, pH 8.0) on a Zeba spin column and concentration to 30 μM. Green fibres at 30 μM were disassembled through the addition of 1 M citrate (0.6 μl of citrate to 20 μl of fibres) to reduce the pH to 3.0. The solution was incubated for 5 min before the addition of Tris (3.6 μl of 1 M stock) to bring the pH back to 8.0; 1 μl of assembled DpHF18–Cy5 fibres at 30 μM was added to the solution. The solution was subsequently incubated at room temperature before centrifugation at 13,000 *g* for 2 min in a benchtop centrifuge. Fibres were resuspended in TBS and imaged by TIRFM.

#### Fibre disassembly

Fast TIRFM imaging of fibres disassembling at low pH was performed on a custom-built TIRF system based on a Nikon Ti stand equipped with perfect focus system alongside a fast *Z* piezo stage (ASI), an azimuthal TIRF illuminator (iLas2, Roper France) with a custom extended field of view (Cairn) and a PLAN Apo 1.45 NA ×100 objective. Images were acquired with a Photometrics Prime 95B back-illuminated sCMOS camera run in pseudo global shutter mode, synchronized with the azimuthal illumination. The system was operated by Metamorph 7.10.1.161. Sulfo-Cy5 maleimide-labelled fibres were imaged with a 630 nm laser (150 mW Coherent OBIS mounted in a Cairn laser launch) and imaged using a Chroma ET655lp filter mounted in a Cairn Optospin wheel at a frame rate of 1 frame every 16 ms.

Fibres were imaged in imaging buffer (25 mM Tris pH 8.0, 100 mM NaCl) in an Ibidi flow cell mounted on clean room-grade coverslips (custom, 25 × 75 mm^2^, Nexterion), and passivated with PLL-PEG (0.1 mg ml^−1^ in 20 mM Hepes, pH 7.6; 5 min). Fibres were allowed to deposit on the coverslip for 5 min before unbound fibres were removed with the imaging buffer. During fast acquisition, the pH was reduced by flowing in low-pH buffer (25 mM Tris, 100 mM NaCl, pH 3.0).

To measure fibre disassembly in bulk solution, pre-formed fibres in 1.5 ml Eppendorf tubes were exchanged into citrate buffers at lower pH to stimulate disassembly. A portion of each pH reaction was removed at various time points and added to a 96 well plate and for 10 min to allow the fibres to settle and adhere to the glass substrate. For each condition and time point, nine fields of view were acquired on an IN Cell Analyzer 2500HS microscope (Molecular Devices) using a Nikon ×60 PLAN Apo 0.95 NA air objective and a 631 nm LED excitation source, 150 ms exposure time with emission collected through a 684 ± 24 nm bandpass filter. Images were quantified using a custom CellProfiler script to segment fibres with the Otsu thresholding algorithm^25^. Upper and lower limits of the threshold, as well as the adaptive window for object ID, were adjusted until fibres were correctly identified relative to the background signal. The major axis length of objects identified using the CellProfiler pipeline were plotted against incubation time for each pH condition.

### Liquid-phase AFM

#### Sample preparation

We incubated 10 µl of a 0.01 wt% poly-lysine solution on a freshly cleaved muscovite mica surface (12 mm, Ted Pella Inc.) for 2 min. The excess solution was removed and the surface was rinsed with water and dried with N_2_ gas^7^. Then 30 µl 10 µM protein solution in the imaging buffer (25 mM Tris-HCl, 400 mM NaCl at pH 8) was incubated on the poly-lysine-coated mica for 30 min and washed with the image buffer to remove excess protein. The pH of the disassembly buffer (25 mM Tris-HCl, 400 mM NaCl, pH 4.1, 4.4, 4, 5 or 4.7) was adjusted with 10 M NaOH or 1 M citric acid and filtered with 0.1 µm pore size PVDF filter before use. For photoacid experiments, 10 µM protein solution in 25 mM Tris-HCl pH 8 was incubated on bare mica for 30 min and washed with 25 mM Tris-HCl pH 5.5; an additional deposition and rinse step was carried out if the number density of fibres on the surface was low. We also freshly prepared 1 mM 2-nitrobenzaldehyde (Sigma-Aldrich) in 25 mM Tris-HCl pH 5.5 and immediately used it without exposure to light at any stage^26^. Spectroscopic and pH measurements indicated that 2-nitrobenzaldehyde is activatable between wavelengths of 200 and 405 nm and lowers the pH from 5.5 to 2.7, and that higher laser intensity leads to faster consumption and acidification.

#### Imaging

For the kinetic study at constant composition, the protein-coated poly-lysine mica substrates were placed under the AFM liquid cell (Bruker Multimode8). Images were captured in the imaging buffer using a clean silicon nitride cantilever (Bruker, SNL-10, spring constant: 0.12 N m^−1^, UV ozoned for 5 min) in tapping mode at room temperature (25 °C). Before flowing the disassembly buffer, the fibres were imaged continuously for 10 min to optimize the parameters (256 scan lines, 1.5 Hz scan rate, high integral gain (3–4) and 50–100 mV free amplitude). After confirming that no cantilever-induced damage occurred, the disassembly buffer was injected continuously at 25 µl min^−1^. The flow-through set-up was optimized to provide negligible residence time and fast pH switching^10^.

For the photoacid study, protein-coated mica with 25 mM Tris-HCl pH 5.5 was placed under the liquid cell of a Cypher VRS AFM (Asylum Research) equipped with BlueDrive laser (×0.3 intensity filter, 405 nm wavelength) with the vent valve open and operated in tapping mode. After confirming the high surface coverage of the fibres, the imaging buffer was replaced with 1 mM 2-nitrobenzaldehyde in 25 mM Tris-HCl pH 5.5, operated without exposure to visible background light and imaged again. The cantilever was then retracted, and BlueDrive was turned on and rastered across pre-selected areas repeatedly using the motorized optical microscope of the AFM. The total UV exposure time during raster/dwell for spot and line patterns was not more than 10 min, after which the cantilever was moved back to the exposed areas and imaged. For global pH changes, the quartz window of the AFM liquid cell in contact with the photoacid solution was exposed to a handheld UV lamp (364 nm wavelength) for 7 min, and then imaged.

Images were processed with Gwyddion SPM v2.62 data analysis software and analysed with Fiji software v1.53s^18^. For kinetics, the total fibre length was measured, and any fragments considered as already disassembled were excluded from the length measurement. To measure the disassembly rate at each end of individual fibres (Supplementary Fig. 8), the centre of the fibre (half of the initial length) was assigned as the second end for measuring length, whereas for fibre fragments, the centre of the fragment was measured as the second end.

## Online content

Any methods, additional references, Nature Portfolio reporting summaries, source data, extended data, supplementary information, acknowledgements, peer review information; details of author contributions and competing interests; and statements of data and code availability are available at 10.1038/s41565-024-01641-1.

### Supplementary information

Supplementary Information Supplementary Figs. 1–12 and Table 1.

Supplementary Data 1 Protein sequences.

## Data Availability

Cryo-EM maps were deposited in the EMDB with the following accession codes: DpHF7, EMD-42075; DpHF18: EMD-42070; DpHF19, EMD-42088. The accompanying structural models were deposited in the PDB with the following accession codes: DpHF7, 8UB3; DpHF18, 8UAO; DpHF19, 8UBG. Protein sequences are available in Supplementary Data 1.

## Code availability

The Rosetta macromolecular modelling suite is available for non-commercial use at https://www.rosettacommons.org. A GitHub repository (https://github.com/shenh2/fiber_design)^27^ contains the Rosetta protocols used to design the protein filaments and the CellProfiler script to quantify fiber lengths.
