# Supplementary material for: De novo design of pH-responsive self-assembling helical protein filaments
Source: Nat Nanotechnol. 2024 Apr 3;19(7):1016–21. doi: 10.1038/s41565-024-01641-1 (PMC11286511; doi:10.1038/s41565-024-01641-1)
Supplement: Supplementary file 1 — Supplementary Figs. 1–12 and Table 1. [file 41565_2024_1641_MOESM1_ESM.pdf]

nature nanotechnology

Article https://doi.org/10.1038/s41565-024-01641-1

De novo design of pH-responsive self-assembling helical protein filaments

In the format provided by the authors and unedited

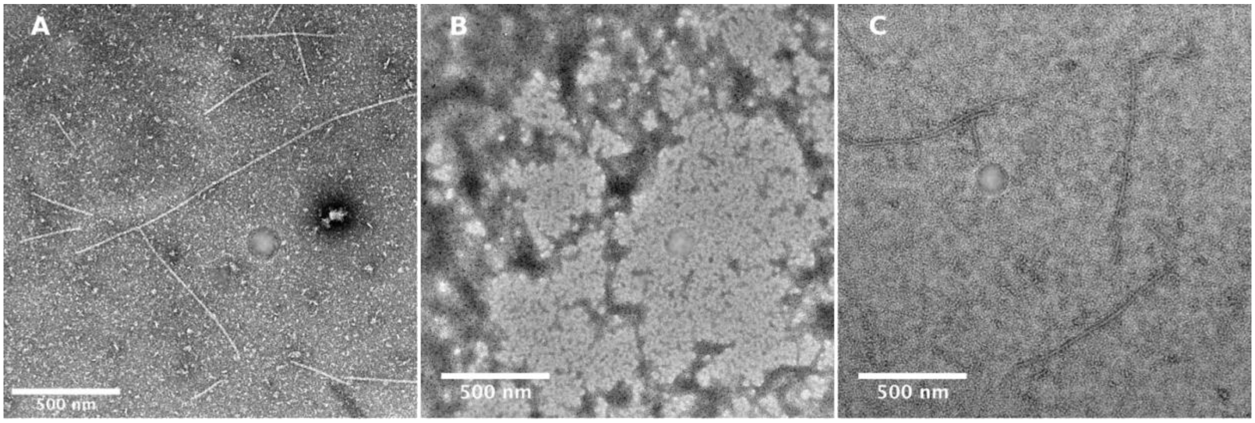

Fig. S1. Reversible assembly of DpHF7 in response to pH. Negative stain EM images for Design DpHF7 (**A**) at pH8, (**B**) drop to pH 4, and (**C**) revert back to pH 8.

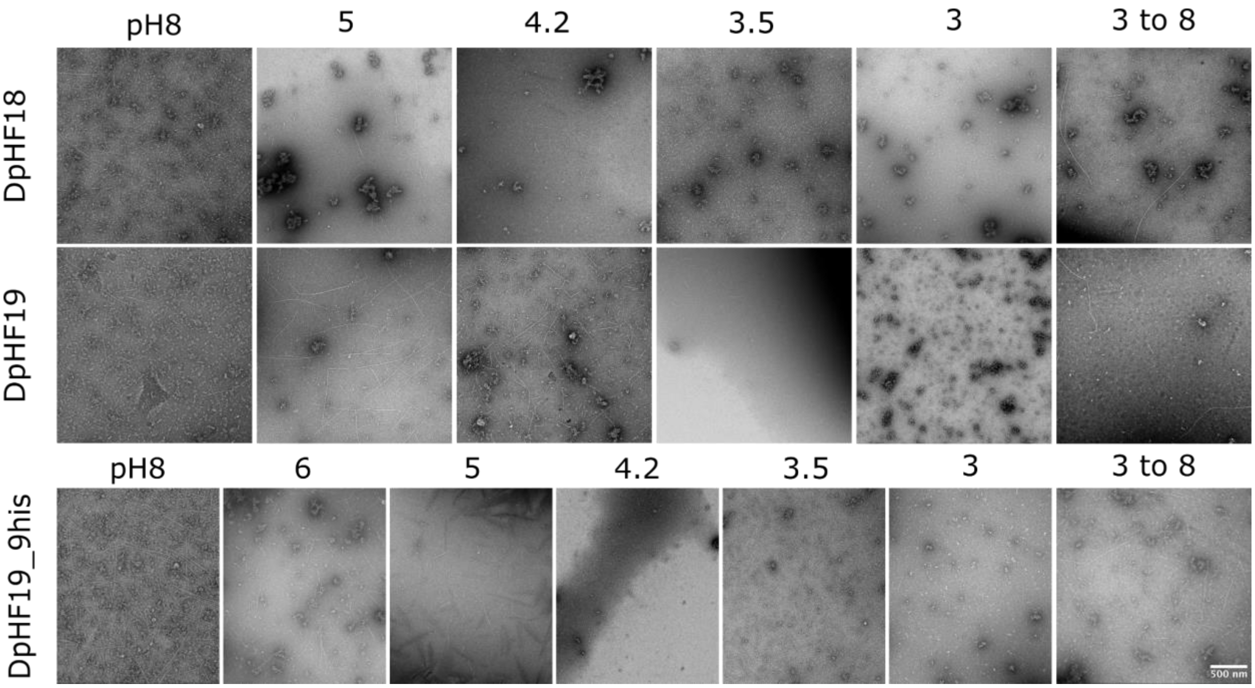

Fig. S2. DpHF18, DpHF19 and DpHF19_9his's assembly state in different pHs characterized by negative stain EM. Representative EM images of DpHF18, DpHF19 and DpHF19_9his in pH 8, 6, 5, 4.2, 3.5, 3 and raised from 3 to 8.

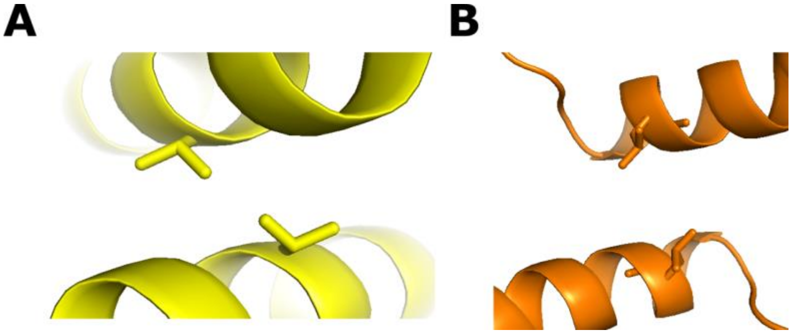

Fig. S3. Hydrophobic residues that lead to the alternating DpHF18 D1 interfaces. (**A**)V29 and (**B**) L82 were mutated to aspartates to knock out the DpHF18 D1 interfaces.

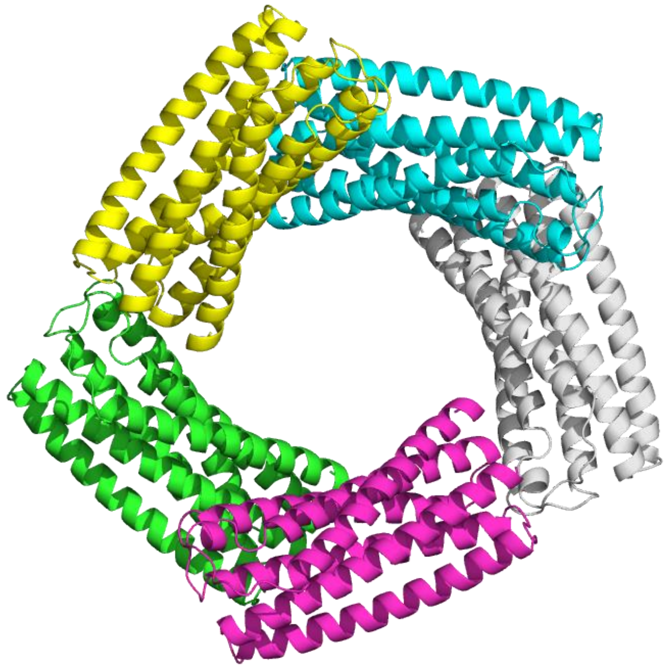

Fig. S4. Cross-section of DpHF19 filament cryoEM structure. Five non-contacting subunits related in the 1-start helical symmetry form a ring.

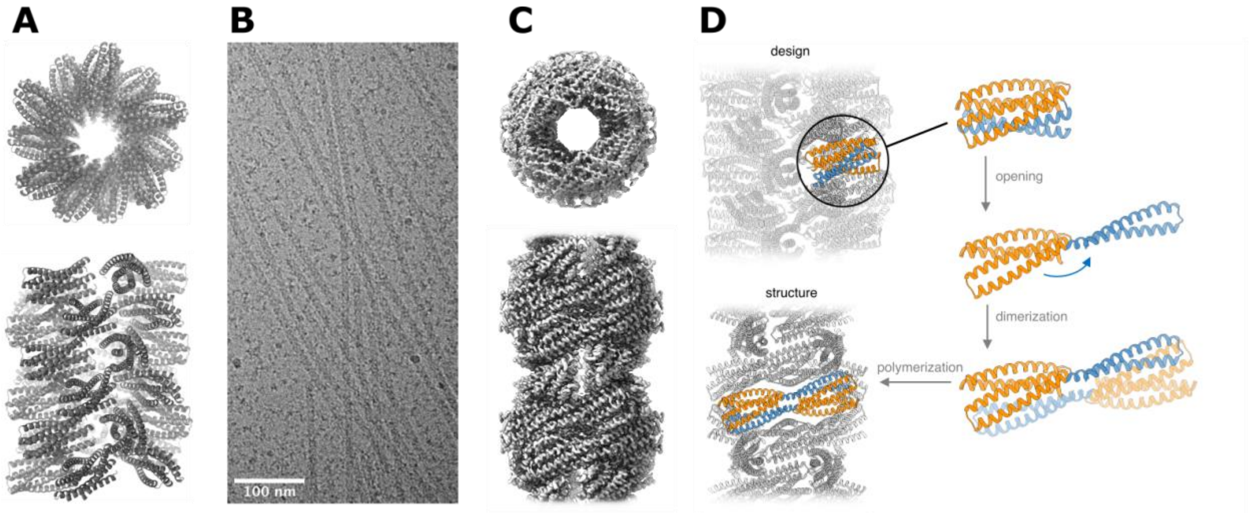

Fig. S5. Deviation of DpHF7 filament cryoEM structure from the design model. (**A**) Computational design model, (**B**) representative filaments in cryoEM micrographs, and (**C**) cryoEM map for DpHF7. (**D**) In the cryoEM structure, two helices flip from the designed monomer and form a dimer, which becomes the helical subunit for the resulting filament.

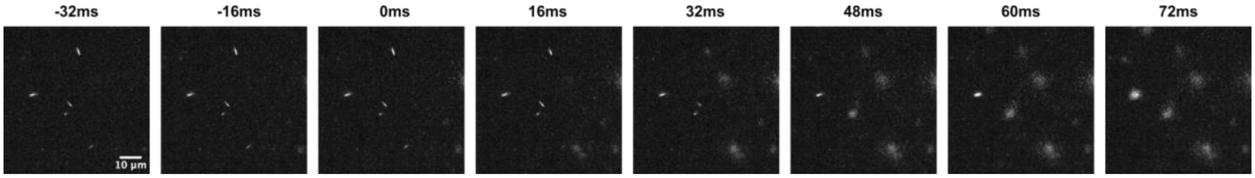

Fig. S6. Rapid disassembly of DpHF18-Cy5 characterized by total internal reflection fluorescent microscopy (TIRFM). Solution pH was dropped from 8 to 3 in a flow cell to image the disassembly of pre-assembled DpHF18-Cy5 fiber. Fibers were stable prior to the addition of citrate, but showed rapid (< 72ms) disassembly concurrent with the reduction of the pH. Citrate was added during imaging, and time 0 was defined as the final frame fibers were stable. We assume that this is the point when pH dropped locally around the fibers (the exact time cannot be controlled with such time resolution), evidenced by the fact that complete disassembly of fibers was seen with 72ms across this field of view (i.e., we don't see stepwise progressive disassembly of fibers; rather we see rapid and complete dissolution).

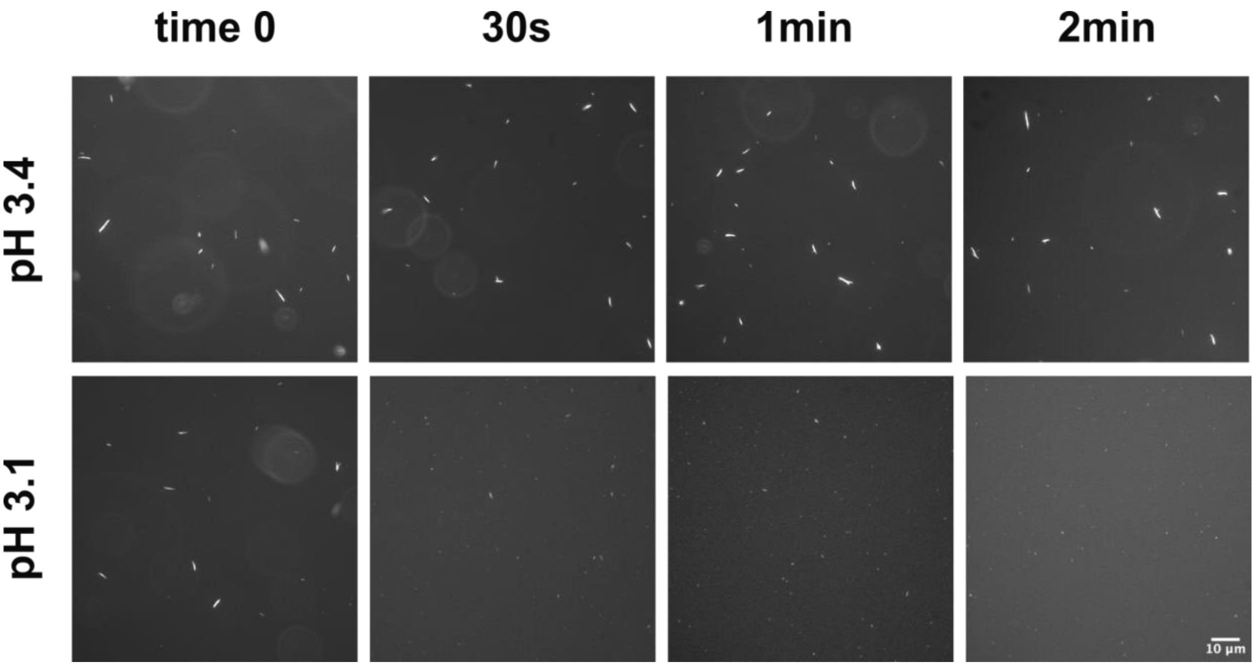

Fig. S7. Sharp pH dependence of assembly. Representative fluorescent microscopy images of DpHF18-Cy5 over 2 minutes following a drop in pH 8.0 to 3.4 or 3.1. Fibers remain assembled at pH 3.4 but disassemble at pH 3.1.

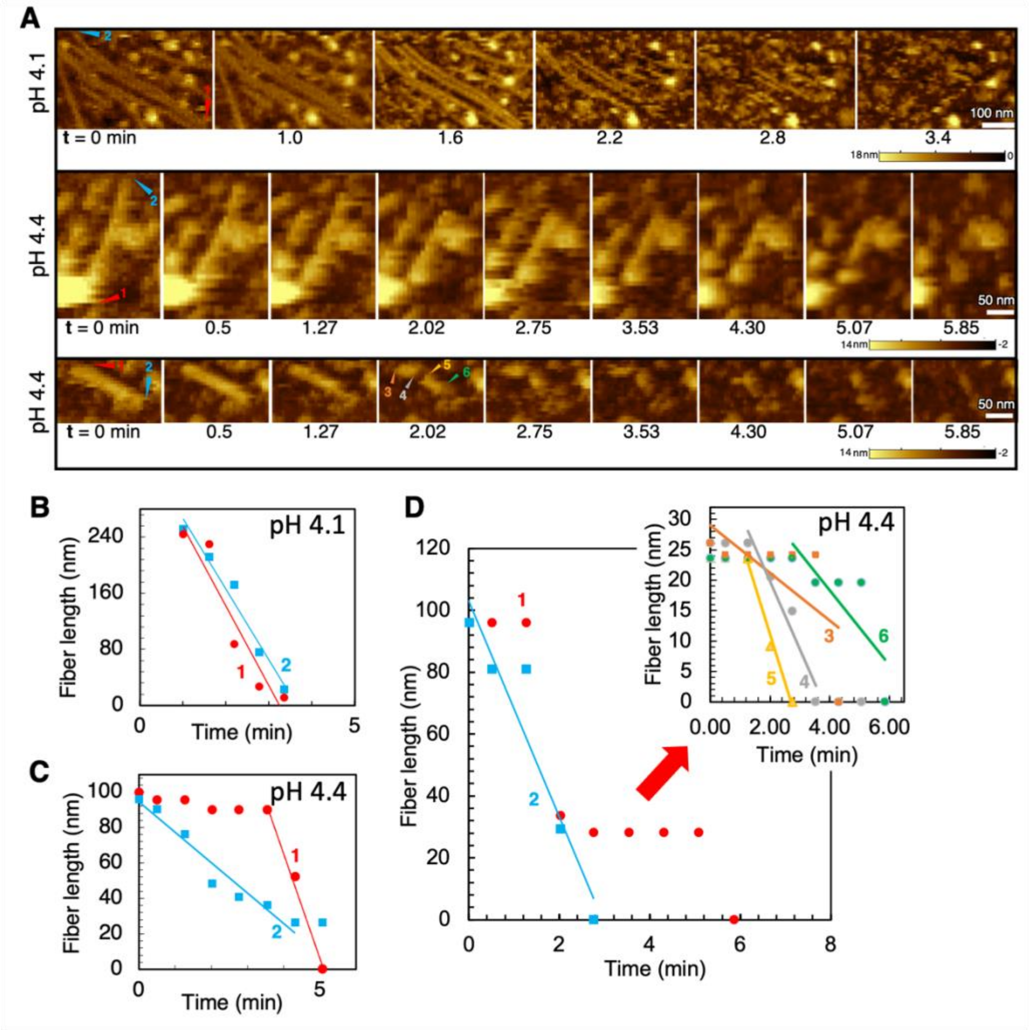

Fig. S8. Disassembly rates from the two ends of individual fibers to the fiber center at pH 4.1 and 4.4. (**A**) Time-lapse of AFM images for DpHF19_9his fibers when pH is reduced from 8 to 4.1 and 4.4. Time at 0 min is defined as the time when the acidic solution is introduced into the flow cell. (**B**-**D**) Comparison of linear fit fiber disassembly rates measured from end 1 (red) and 2 (blue) to center of fibers shown in (A) at pH 4.1 and 4.4. Data shows different mechanisms and rates of disassembly. (**B**) The fiber disassembles at nearly the same rate from ends 1 and 2. (**C**) End 1 of the fiber incurs a delay prior to constant disassembly, while end 2 disassembles constantly from 0.5 min. (**D**) End 2 constantly disassembles, while end 1 does not initially disassemble but fragments at 2.02 min and further disassembles at ends 3, 4, 5, and 6 with their unique disassembly rates, as shown in the inset plot.

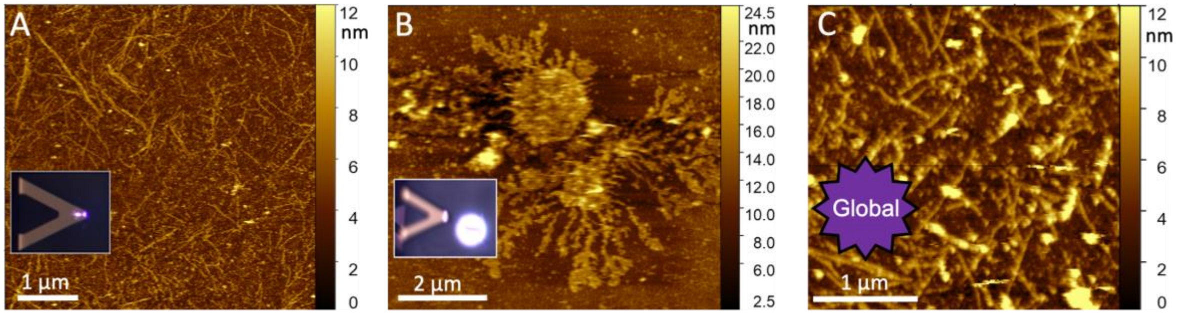

Fig. S9. Control experiments using UV with and without photoacid on deposited DpHF19_9his fibers show fiber disassembly is driven by acidifying the local environment rather than UV-induced denaturation. (**A**) Simultaneous imaging and exposure of fibers to the laser (405 nm, 0.3 x filter) without photoacid in solution shows fibers are intact. The inset demonstrates the method wherein the laser is partially positioned on the AFM cantilever for photothermal excitation for imaging while the surface is exposed to the rest. (**B**) Overexposure of fibers under photoacid to higher intensity laser (1x filter) shows fibers aggregated and lost their structure, likely due to local heating rather than disassembly. The inset shows a large intense laser spot that was imaged after exposure. (**C**) Fibers were unaffected after the laser patterned sample surface with consumed photoacid solution (Fig. 4e) was exposed to a global UV source (handheld lamp, 364 nm) for 7 min.

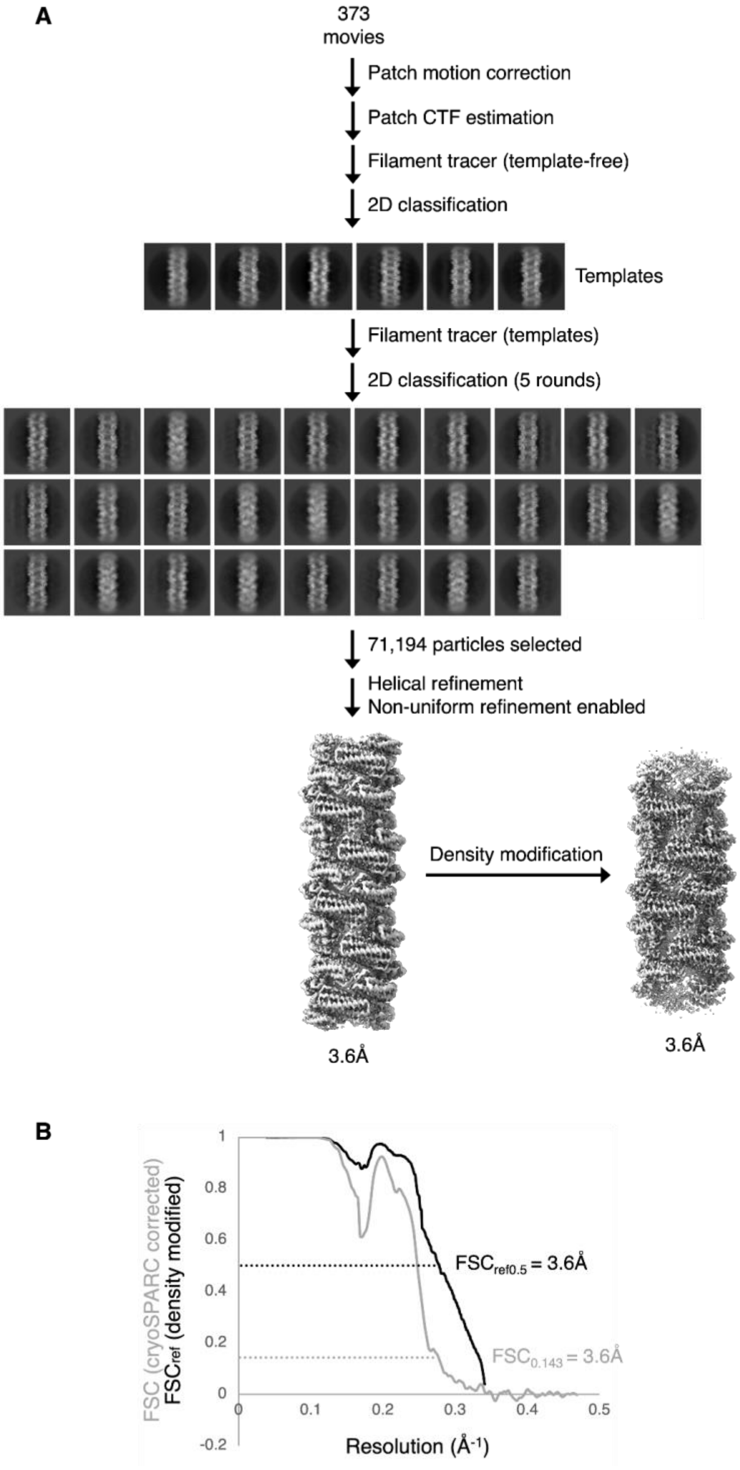

Fig. S10. Cryo-EM data processing for DpHF18. (**A**) Flowchart of cryo-EM data processing. (**B**) Noise-substituted corrected FSC curve from cryoSPARC (grey) and FSC_{ref} curve after density modification (black) and corresponding resolution estimates.

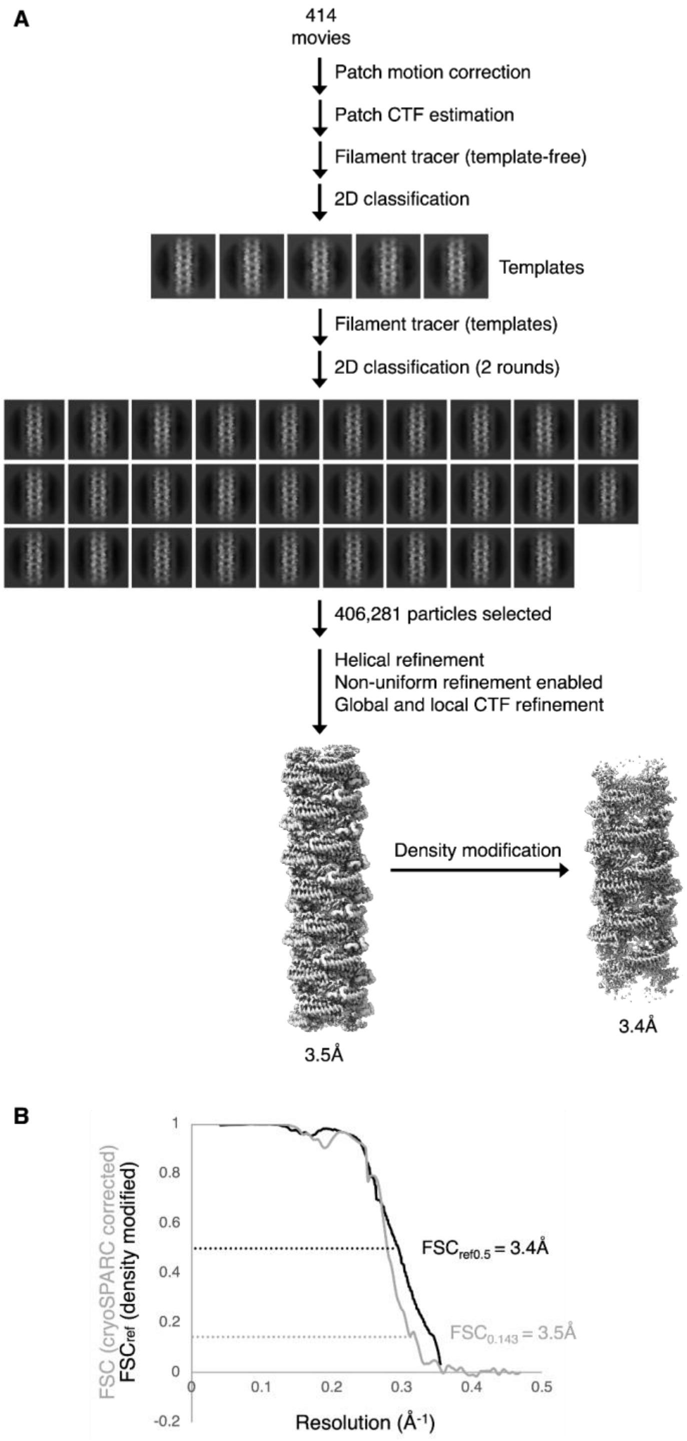

Fig. S11. Cryo-EM data processing for DpHF19. (**A**) Flowchart of cryo-EM data processing. (**B**) Noise-substituted corrected FSC curve from cryoSPARC (grey) and FSC_{ref} curve after density modification (black) and corresponding resolution estimates.

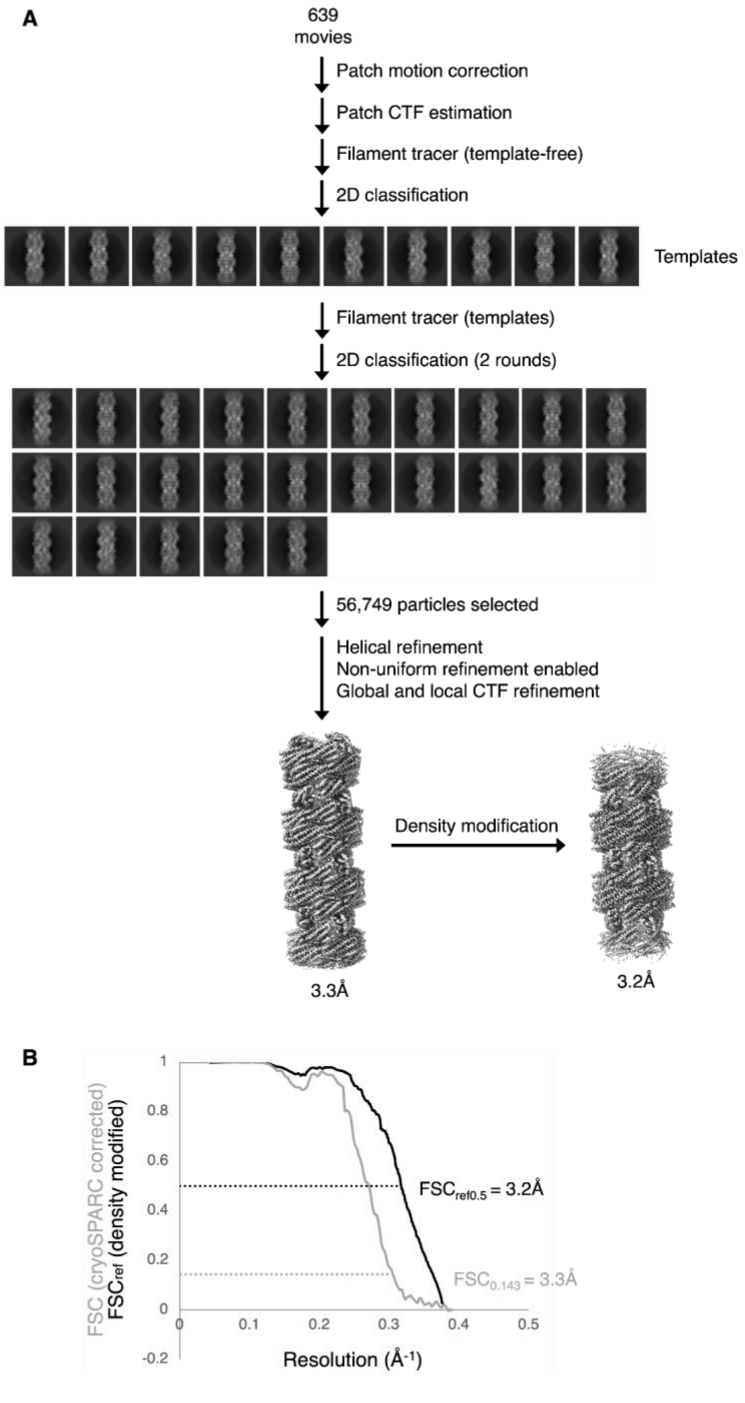

Fig. S12. Cryo-EM data processing for DpHF7. (**A**) Flowchart of cryo-EM data processing. (**B**) Noise-substituted corrected FSC curve from cryoSPARC (grey) and FSC_{ref} curve after density modification (black) and corresponding resolution estimates.

Table S1: Cryo-EM data collection, refinement and validation statistics

	DpHF7	DpHF18	DpHF19
**PDB code**	8UB3	8UAO	8UBG
**EMDB code**	42075	42070	42088
**Magnification**	130000	130000	36000
**Voltage (kV)**	300	300	200
**Electron fluence (e–/Å2)**	90	90	65
**Defocus range (μm)**	0.5-2	0.5-2	0.5-2.4
**Pixel size (data collection) (Å)**	0.525	0.525	1.16
**Pixel size (reconstruction) (Å)**	1.05	1.05	1.16
**Point group symmetry**	D2	D1	C1
**Helical rise (Å)**	24	16.7	8.4
**Helical rotation (degrees)**	-43.1	59.3	-148.9
**Particle images (no.)**	56,749	71,194	406,281
**Resolution (0.143 FSC) (Å)**	3.3	3.6	3.5
**Resolution, density modified (0.5 FSCref) (Å)**	3.2	3.6	3.4
**R.m.s. deviation bond lengths (Å)**	0.3	0.28	0.29
**R.m.s. deviation bond angles (°)**	0.53	0.52	0.56
**MolProbity score**	1.23	1.04	0.91

**Clashscore**	4.56	3	2
**C-beta deviations**	0	0	0
**Rotamer outliers (%)**	0	0	0
**Ramachandran favored (%)**	100	100	99.5
**Ramachandran allowed (%)**	0	0	0.5
**Ramachandran outliers (%)**	0	0	0
